# China’s regional inequity in pharmacist’s drug safety practice

**DOI:** 10.1186/1475-9276-11-38

**Published:** 2012-08-06

**Authors:** Liyang Tang

**Affiliations:** 1 Department of Economics, School of Economics and Management, Tsinghua University, Beijing, 100084, China

## Abstract

**Background:**

The promotion of patient safety and drug safety through promotion of pharmacist’s drug safety practice was among the most important aims of China’s health delivery system reform, but regional inequity in pharmacist’s drug safety practice was still serious in China.

**Methods:**

The 2011 national patient safety and medication error baseline survey was carried out for the first time in China, and through analyzing dataset from the survey, this study was set up to test both China’s regional inequity in pharmacist’s drug safety practice and major influencing factors for pharmacist’s drug safety practice among different districts of China.

**Results:**

Pharmacist’s drug safety practice in regions with higher per capita GDP and more abundant medical resources was still better than that in regions with lower per capita GDP and less abundant medical resources. In all districts of China, pharmacist’s drug safety knowledge, drug safety attitude, self-perceived pressure and fatigue, hospital management quality, and hospital regulation were major influencing factors for pharmacist’s drug safety practice, while only in regions with higher per capita GDP and more abundant medical resources, hospital drug safety culture, supervisor’s work team management, cooperation atmosphere of work team, and drug safety culture of work team were major influencing factors for pharmacist’s drug safety practice.

**Conclusion:**

Regional inequity in pharmacist’s drug safety practice still existed in China. In all districts of China, promoting pharmacist’s drug safety knowledge, drug safety attitude, self-perceived pressure and fatigue, hospital management quality, and hospital regulation could help promote pharmacist’s drug safety practice, while only in regions with higher per capita GDP and more abundant medical resources, promoting hospital drug safety culture, supervisor’s work team management, cooperation atmosphere of work team, and drug safety culture of work team could help promote pharmacist’s drug safety practice. And in regions with lower per capita GDP and less abundant medical resources, the link between pharmacist’s drug safety practice and hospital drug safety culture/supervisor’s work team management/cooperation atmosphere of work team/drug safety culture of work team should also be gradually established.

## Background

The promotion of patient safety and drug safety through promotion of pharmacist’s drug safety practice was one of the most important aims of China’s health delivery system reform [1]. At the national level, Ministry of Health, State Administration of Traditional Chinese Medicine, and State Food and Drug Administration issued many policies on strengthening drug safety management in pharmacy, improving drug safety related pharmacy service quality, and establishing/improving control system for the management of drug safety related pharmacy service quality, in the project of Hospital Management Year pharmacist’s drug safety practice was also especially emphasized [2-8]. Although these policies and measures promoted pharmacist’s drug safety consciousness and improved drug safety related pharmacy service quality to a certain degree all around China, due to serious regional inequity in economic resources and medical resources, the attention paid to the promotion of patient safety and drug safety through promotion of pharmacist’s drug safety practice varied across regions [9]. In fact China’s regional inequity in both economic resources and medical resources was really serious because of a vicious cycle of three synergistic reasons: regional market failure and insufficient regional government stewardship, regional inequitable distribution of social determinants of economic resources/medical resources, and regional public dissatisfaction with the fairness and trustworthiness of economic/medical resource allocation system [10]. In this context, due to different degrees of regional market failure and different levels of regional government management capacity, different degrees of secondary distribution adjustment for regional inequitable distribution of social determinants of economic resources/medical resources, and different degrees of regional public confidence in the fairness and trustworthiness of economic/medical resource allocation system among different districts of China, the efforts in promoting patient safety and drug safety through promoting pharmacist’s drug safety practice were significantly different among regions [10-15], and regions with higher per capita GDP and more abundant medical resources usually performed better on the promotion of patient safety and drug safety through promotion of pharmacist’s drug safety practice than regions with lower per capita GDP and less abundant medical resources [9]. As researchers have already pointed out, regional inequity in pharmacist’s drug safety practice was still serious in China [16-22].

Many previous studies separately found that pharmacist’s drug safety knowledge, drug safety attitude, and drug safety environment (involving facility and equipment, regulation, personal pressure and fatigue, hospital management quality, hospital drug safety culture, and drug safety culture of work team) were major influencing factors for pharmacist’s drug safety practice both at the national level and at the regional level [23-39], but the regional differences in major influencing factors for pharmacist’s drug safety practice haven’t been studied under a systematic and comprehensive framework, and then the relative importance of influencing factors for pharmacist’s drug safety practice among different districts of China wasn’t clear [9].

Some previous studies further subdivided pharmacist’s drug safety practice into detailed aspects, most of them only focused on pharmacist’s basic drug safety practice (including drug management, pharmacist’s dispensing behavior, and medication error recording and reporting) and its major influencing factors, while little study focused on pharmacist’s advanced drug safety practice (including medication error analysis, sharing and correction, and implementation of drug safety system and measure) and its major influencing factors [9,23-27,29,30,32,33,35-39]. And there was no previous study which explored regional differences in major influencing factors for detailed aspects of pharmacist’s drug safety practice in China [9].

Besides shortcomings and deficiencies mentioned above, there were several other serious problems with these previous studies: most of these studies obtained their conclusions only through qualitative analysis and authors’ experiences, while little study adopted strict quantitative analysis, and their conclusions were usually drawn on the basis of a small range of pharmacists, then the robustness and universality of their conclusions remained controversial [23,24,26-32,34-39].

### Aim of the study

Under the background of China’s health delivery system reform, in order to find the most effective ways to promote patient safety and drug safety through promoting pharmacist’s drug safety practice among different districts of China, this study first carried out the 2011 national patient safety and medication error baseline survey, and then through analyzing dataset from the survey, this study tested both China’s regional inequity in pharmacist’s drug safety practice and major influencing factors for pharmacist’s drug safety practice among different districts of China. On the basis of main findings, inspirations on regional differences in effective ways to promote patient safety and drug safety through promoting pharmacist’s drug safety practice were also found for China’s future health delivery system reform.

## Methods

### Data

The 2011 national patient safety and medication error baseline survey was carried out by the patient safety and drug safety research group (this research group consisted of the pharmacy department of Peking University Third Hospital, the science and technology development center of Chinese Pharmaceutical Association, and Tsinghua University). Through the stratified sampling method, 126 hospitals from 26 provinces, autonomous regions, and municipalities directly under the central government were chosen, and among them 112 hospitals responded to this survey (the response rate was 88.89%). 5844 pharmacists in these 112 hospitals were chosen through the stratified sampling design, and among them 1959 pharmacists completed the questionnaire with assistance from the patient safety and drug safety research group (the rate of valid questionnaire was 33.52%).

Questionnaire for the patient safety and medication error baseline survey was designed on the basis of questionnaire for hospital survey on patient safety culture [40], patient safety/drug safety situation in China, and pharmacy experts’ experiences, it contained four parts: the first part inquired about pharmacist’s drug safety knowledge (including 24 questions), the second part inquired about pharmacist’s drug safety attitude (including 16 questions), the third part inquired about pharmacist’s drug safety environment (including 28 questions), and the fourth part inquired about pharmacist’s drug safety practice (including 34 questions). Pharmacist’s drug safety environment and drug safety practice were further subdivided into detailed aspects: drug safety environment was composed of the aspect of physical work environment, the aspect of self-perceived pressure and fatigue, the aspect of hospital management quality, the aspect of hospital regulation, the aspect of hospital drug safety culture, the aspect of supervisor’s work team management, the aspect of cooperation atmosphere of work team, and the aspect of drug safety culture of work team; drug safety practice was composed of the aspect of drug management, the aspect of pharmacist’s prescription dispensing behavior, the aspect of standardization of physician’s prescription (special aspect since it needed the cooperation of doctor and pharmacist), the aspect of medication error recording and reporting, the aspect of medication error analysis, sharing and correction, and the aspect of implementation of drug safety system and measure. At the beginning of questionnaire, there were detailed explanations for professional nouns in the following questions, and at the end of questionnaire, pharmacists were asked for their personal attribute information involving personal average working load (daily processing of prescription/doctor’s advice), personal professional title, and personal professional position. 112 hospitals which responded to this survey were asked for hospital attribute information involving hospital level, length of hospital history, and hospital type. All these selected personal attributes and hospital attributes were considered as possible influencing factors for pharmacist’s drug safety practice by the research group, they were split into dummy variables and were mainly treated as control variables in regression analysis, and their detailed explanations could be found in Table 1.

**Table 1:** Descriptive statistics of personal attributes and hospital attributes

**Dummy variables**	**Descriptions**	**Mean**	**Standard deviation**
Personal average working load dummy variables	1 = The number of prescriptions/doctor’s advices processed per day is 0, 0 = Otherwise	0.163	0.370
	1 = The number of prescriptions/doctor’s advices processed per day is between 1 and 100, 0 = Otherwise	0.247	0.432
	1 = The number of prescriptions/doctor’s advices processed per day is between 101 and 300, 0 = Otherwise	0.249	0.432
	1 = The number of prescriptions/doctor’s advices processed per day is between 301 and 500, 0 = Otherwise	0.161	0.367
	1 = The number of prescriptions/doctor’s advices processed per day is more than 500, 0 = Otherwise	0.180	0.385
Personal professional title dummy variables	1 = Practice pharmacist, 0 = Otherwise	0.035	0.185
	1 = Assistant pharmacist (junior title), 0 = Otherwise	0.112	0.315
	1 = Pharmacist (junior title), 0 = Otherwise	0.360	0.480
	1 = Competent pharmacist (intermediate title), 0 = Otherwise	0.325	0.469
	1 = Associate senior pharmacist (senior title), 0 = Otherwise	0.125	0.331
	1 = Senior pharmacist (senior title), 0 = Otherwise	0.042	0.202
Personal professional position dummy variables	1 = Drug dispensing position in outpatient pharmacy/emergency pharmacy, 0 = Otherwise	0.456	0.498
	1 = Drug dispensing position in inpatient pharmacy, 0 = Otherwise	0.178	0.382
	1 = Position in drug storehouse, 0 = Otherwise	0.082	0.275
	1 = Hospital preparation production position, 0 = Otherwise	0.042	0.201
	1 = Position in intravenous compounding center, 0 = Otherwise	0.058	0.235
	1 = Clinical pharmacy position, 0 = Otherwise	0.133	0.340
	1 = Other position for part-time job, 0 = Otherwise	0.006	0.075
	1 = Other position, 0 = Otherwise	0.044	0.206
Hospital level dummy variables	1 = Sample pharmacist is in Second-Class Hospital at Grade 2, 0 = Otherwise	0.003	0.055
	1 = Sample pharmacist is in First-Class Hospital at Grade 2, 0 = Otherwise	0.097	0.297
	1 = Sample pharmacist is in Second-Class Hospital at Grade 3, 0 = Otherwise	0.092	0.289
	1 = Sample pharmacist is in First-Class Hospital at Grade 3, 0 = Otherwise	0.808	0.394
Length of hospital history dummy variables	1 = Sample pharmacist is in hospital established before the founding of the People’s Republic of China, 0 = Otherwise	0.429	0.495
	1 = Sample pharmacist is in hospital established after the founding of the People’s Republic of China, 0 = Otherwise	0.493	0.500
Hospital type dummy variables	1 = Sample pharmacist is in traditional Chinese medicine specialized hospital, 0 = Otherwise	0.002	0.045
	1 = Sample pharmacist is in western medicine specialized hospital, 0 = Otherwise	0.078	0.268
	1 = Sample pharmacist is in traditional Chinese medicine general hospital, 0 = Otherwise	0.033	0.179
	1 = Sample pharmacist is in integrated traditional and western medicine general hospital, 0 = Otherwise	0.022	0.146
	1 = Sample pharmacist is in western medicine general hospital, 0 = Otherwise	0.865	0.341

The use of the dataset was approved by the science and technology development center of Chinese Pharmaceutical Association.

### Regional grouping according to regional economic resources/medical resources

**Figure 1:**
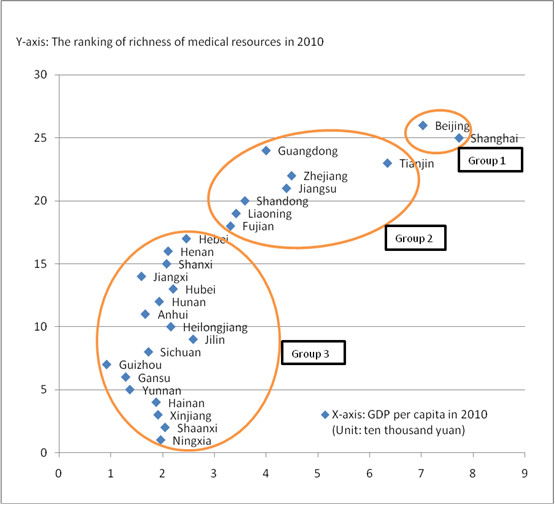
Regional grouping result.

In this study, according to the regional per capita GDP and the ranking of richness of regional medical resources (calculated based on per capita medical service resources and per capita medical personnel resources in level 3 hospitals/level 2 hospitals) in 2010 (data in 2010 was chosen because the 2011 national patient safety and medication error baseline survey mainly collected information in 2010) [9], 26 provinces, autonomous regions, and municipalities directly under the central government were divided into 3 groups through clustering analysis (presented in Figure 1):

1. Regions with the highest per capita GDP and the most abundant medical resources (Group 1): Beijing, Shanghai;

2. Regions with the second highest per capita GDP and the second most abundant medical resources (Group 2): Guangdong, Tianjin, Zhejiang, Jiangsu, Shandong, Liaoning, Fujian;

3. Regions with the lowest per capita GDP and the least abundant medical resources (Group 3): Hebei, Henan, Shanxi, Jiangxi, Hubei, Hunan, Anhui, Heilongjiang, Jilin, Sichuan, Guizhou, Gansu, Yunnan, Hainan, Xinjiang, Shaanxi, Ningxia.

### Measures of pharmacist’s drug safety knowledge, drug safety attitude, drug safety environment, and drug safety practice

The 5-item self-reporting measure that assessed each question in the questionnaire on a scale of 1 to 5 was employed, higher score reflected better performance in the question: the option “excellent performance” was assigned score 5, the option “good performance” was assigned score 4, and the option “general performance” was assigned score 3, the option “bad performance” was assigned score 2, and the option “poor performance” was assigned score 1.

Pharmacist’s drug safety knowledge, drug safety attitude, drug safety environment/detailed aspects of drug safety environment, and drug safety practice/detailed aspects of drug safety practice were all composed of several questions in the questionnaire, and the weighted average score of corresponding question set was the overall score for pharmacist’s drug safety knowledge/drug safety attitude/drug safety environment or its detailed aspect/drug safety practice or its detailed aspect.

### Regression model

In order to test major influencing factors for pharmacist’s drug safety practice among different districts of China, the following ordinary least squares (OLS) regression model was separately estimated for different regional groups:

(1) $${\text{practice}}_{i}=\beta_{0}+\beta_{1}{\text{knowledge}}_{i}+\beta_{2}{\text{attitude}}_{i}+\sum_{n}\beta_{n3}{\text{environment}}_{\text{ni}}+\sum_{p}\beta_{p4}z_{\text{pi}}+\epsilon_{\text{i}}\left(1\right)$$

here i was sample number; practice_i_ was drug safety practice; knowledge_i_ was drug safety knowledge; attitude_i_ was drug safety attitude; environment_ni_ (n = 1,2,…,8) were detailed aspects of drug safety environment; z_pi_ were control variables, since selected personal attributes (involving personal average working load, personal professional title, and personal professional position) and hospital attributes (involving hospital level, length of hospital history, and hospital type) may be key influencing factors for pharmacist’s drug safety practice, they were all controlled as dummy variables in the regression model; error term ϵ_i_ was assumed to be distributed normal.

In order to test major influencing factors for detailed aspects of pharmacist’s drug safety practice among different districts of China, the following six OLS regression models (corresponding to q = 1,2,…,6) were separately estimated for different regional groups:

(2) $${\text{practice}}_{\text{qi}}=\beta_{0}+\beta_{1}{\text{knowledge}}_{i}+\beta_{2}{\text{attitude}}_{i}+\sum_{n}\beta_{n3}{\text{environment}}_{\text{ni}}+\sum_{\text{p}}\beta_{p4}z_{\text{pi}}+\epsilon_{i}\left(2\right)-\left(7\right)$$

here practice_qi_ (q = 1,2,…,6) were detailed aspects of drug safety practice; descriptions of other variables were the same as those in regression model (1).

## Results

### Descriptive statistics

The descriptive statistics of pharmacist’s drug safety knowledge, drug safety attitude, detailed aspects of drug safety environment, drug safety practice, and detailed aspects of drug safety practice were presented in Table 2. From the descriptive statistics of drug safety practice/detailed aspects of drug safety practice, regional inequity in pharmacist’s drug safety practice still existed in China, and specifically speaking, regional inequity in all detailed aspects of pharmacist’s drug safety practice still existed in China. From the descriptive statistics of major influencing factors for pharmacist’s drug safety practice, regional inequity in pharmacist’s drug safety knowledge, drug safety attitude, and detailed aspects of drug safety environment still existed in China.

**Table 2:** Descriptive statistics of pharmacist’s drug safety knowledge, drug safety attitude, drug safety environment, and drug safety practice

	**Group 1 (N = 777)**	**Group 2 (N = 511)**	**Group 3 (N = 671)**			
**Variable**	**Mean**	**Mean**	**Mean**	**Standard deviation**	**Min**	**Max**
Drug safety knowledge	3.462	3.454	3.444	0.226	1	5
Drug safety attitude	3.758	3.722	3.690	0.452	1	5
Physical work environment	3.327	3.291	3.110	0.810	1	5
Self-perceived pressure and fatigue	3.373	3.242	3.221	0.851	1	5
Hospital management quality	2.809	2.716	2.603	1.144	1	5
Hospital regulation	4.014	3.965	3.951	0.462	1	5
Hospital drug safety culture	3.395	3.378	3.325	0.370	1	5
Supervisor’s work team management	3.771	3.747	3.697	0.601	1	5
Cooperation atmosphere of work team	3.693	3.674	3.619	0.501	1	5
Drug safety culture of work team	3.154	3.153	3.140	0.372	1	5
Drug safety practice	3.753	3.659	3.658	0.482	1	5
Drug management	4.110	4.084	4.059	0.467	1	5
Pharmacist’s prescription dispensing behavior	3.949	3.817	3.761	0.519	1	5
Standardization of physician’s prescription	3.545	3.495	3.314	0.632	1	5
Medication error recording and reporting	3.681	3.595	3.557	1.045	1	5
Medication error analysis, sharing and correction	3.666	3.573	3.537	0.824	1	5
Implementation of drug safety system and measure	3.659	3.603	3.581	0.544	1	5

### Regression results on pharmacist’s drug safety practice

Results of regression model (1) were presented in Table 3. From the significance and size of the coefficient for drug safety knowledge/drug safety attitude/detailed aspect of drug safety environment, major influencing factors for pharmacist’s drug safety practice among different districts of China were revealed as follows.

**Table 3:** Results of regression model (1)

	**(1)**	**(2)**	**(3)**
	**Drug safety practice**		
	**Group 1**	**Group 2**	**Group 3**
Drug safety knowledge	0.228***	0.223***	0.218***
	(3.62)	(3.01)	(3.10)
Drug safety attitude	0.263***	0.126***	0.118**
	(7.19)	(2.93)	(2.40)
Physical work environment	0.00614	0.0204	0.0367
	(0.32)	(0.70)	(1.52)
Self-perceived pressure and fatigue	0.0788***	0.102***	0.110***
	(2.76)	(2.61)	(2.96)
Hospital management quality	0.0408*	0.0480*	0.0538*
	(1.91)	(1.79)	(1.81)
Hospital regulation	0.318***	0.393***	0.432***
	(9.25)	(8.34)	(10.53)
Hospital drug safety culture	0.134**	0.111**	0.0775
	(2.40)	(2.06)	(1.13)
Supervisor’s work team management	0.0887***	0.0453	0.0367
	(2.83)	(1.12)	(0.99)
Cooperation atmosphere of work team	0.258***	0.0118	0.0488
	(5.41)	(0.36)	(1.11)
Drug safety culture of work team	0.306***	0.258***	0.00809
	(5.16)	(4.48)	(0.20)
Personal average working load dummy variables	Yes	Yes	Yes
Personal professional title dummy variables	Yes	Yes	Yes
Personal professional position dummy variables	Yes	Yes	Yes
Hospital level dummy variables	Yes	Yes	Yes
Length of hospital history dummy variables	Yes	Yes	Yes
Hospital type dummy variables	Yes	Yes	Yes
Constant	−0.641	−0.399	−0.896**
	(−1.47)	(−1.02)	(−2.48)
N	1959	1959	1959
R^2^	0.614	0.688	0.638

*t* statistics in parentheses, * *p* < 0.10,** *p* < 0.05,*** *p* < 0.01.

In group 1, the most important influencing factors for pharmacist’s drug safety practice were hospital regulation and drug safety culture of work team, and the second most important influencing factors for pharmacist’s drug safety practice were pharmacist’s drug safety attitude, cooperation atmosphere of work team, and pharmacist’s drug safety knowledge, while hospital drug safety culture, supervisor’s work team management, self-perceived pressure and fatigue, and hospital management quality also had significant positive influences on pharmacist’s drug safety practice, but physical work environment had no influence on pharmacist’s drug safety practice.

In group 2, the most important influencing factor for pharmacist’s drug safety practice was hospital regulation, and the second most important influencing factors for pharmacist’s drug safety practice were drug safety culture of work team and pharmacist’s drug safety knowledge, while pharmacist’s drug safety attitude, hospital drug safety culture, self-perceived pressure and fatigue, and hospital management quality also had significant positive influences on pharmacist’s drug safety practice, but physical work environment, supervisor’s work team management, and cooperation atmosphere of work team had no influence on pharmacist’s drug safety practice.

In group 3, the most important influencing factor for pharmacist’s drug safety practice was hospital regulation, and the second most important influencing factor for pharmacist’s drug safety practice was pharmacist’s drug safety knowledge, while pharmacist’s drug safety attitude, self-perceived pressure and fatigue, and hospital management quality also had significant positive influences on pharmacist’s drug safety practice, but physical work environment, hospital drug safety culture, supervisor’s work team management, cooperation atmosphere of work team, and drug safety culture of work team had no influence on pharmacist’s drug safety practice.

### Regression results on detailed aspects of pharmacist’s drug safety practice

Results of regression model (2)-(7) were correspondingly presented in Table 4 ,Table 5, and Table 6. From the significance and size of the coefficient for drug safety knowledge/drug safety attitude/detailed aspect of drug safety environment, major influencing factors for detailed aspects of pharmacist’s drug safety practice among different districts of China were revealed as follows.

**Table 4:** Results of regression model (2)-(3)

	**(1)**	**(2)**	**(3)**	**(4)**	**(5)**	**(6)**
	**Drug management**			**Pharmacist’s prescription dispensing behavior**		
	**Group 1**	**Group 2**	**Group 3**	**Group 1**	**Group 2**	**Group 3**
Drug safety knowledge	0.340***	0.268***	0.219***	0.405***	0.364***	0.294***
	(5.09)	(3.40)	(2.79)	(3.84)	(3.76)	(3.27)
Drug safety attitude	0.0590	0.0527	0.0178	0.241***	0.213***	0.151**
	(0.63)	(1.09)	(0.39)	(4.63)	(3.83)	(2.33)
Physical work environment	0.00729	0.0338	0.0238	−0.0265	−0.0170	−0.0172
	(0.36)	(1.18)	(0.93)	(−0.97)	(−0.44)	(−0.55)
Self-perceived pressure and fatigue	0.0173	0.0456	0.0543	0.0697*	0.0739**	0.112***
	(0.45)	(0.88)	(0.78)	(1.77)	(2.44)	(2.76)
Hospital management quality	0.0302*	0.0509**	0.0701***	0.0371**	0.0710**	0.132**
	(1.94)	(2.25)	(2.78)	(2.12)	(2.30)	(2.48)
Hospital regulation	0.563***	0.579***	0.638***	0.236***	0.268***	0.400***
	(15.48)	(12.53)	(14.66)	(3.79)	(5.47)	(7.49)
Hospital drug safety culture	0.0454	0.0425	0.0196	0.141***	−0.0737	0.0867
	(0.80)	(0.63)	(0.33)	(2.64)	(−0.81)	(1.20)
Supervisor’s work team management	0.0465	0.0472	0.0149	0.125*	−0.0249	−0.00480
	(1.40)	(1.20)	(0.38)	(1.67)	(−0.56)	(−0.10)
Cooperation atmosphere of work team	0.0289	0.0393	0.0196	0.202***	0.0369	0.0372
	(0.84)	(0.91)	(0.47)	(2.64)	(0.64)	(0.72)
Drug safety culture of work team	0.130*	−0.00236	−0.0513	0.294***	0.151***	0.103
	(1.90)	(−0.04)	(−0.84)	(3.75)	(2.97)	(1.38)
Personal average working load dummy variables	Yes	Yes	Yes	Yes	Yes	Yes
Personal professional title dummy variables	Yes	Yes	Yes	Yes	Yes	Yes
Personal professional position dummy variables	Yes	Yes	Yes	Yes	Yes	Yes
Hospital level dummy variables	Yes	Yes	Yes	Yes	Yes	Yes
Length of hospital history dummy variables	Yes	Yes	Yes	Yes	Yes	Yes
Hospital type dummy variables	Yes	Yes	Yes	Yes	Yes	Yes
Constant	0.00810	0.355	0.379	1.294**	0.622	−0.415
	(0.02)	(0.93)	(1.03)	(2.08)	(1.20)	(−0.92)
N	777	511	671	777	511	671
R^2^	0.554	0.653	0.585	0.343	0.477	0.462

*t* statistics in parentheses, * *p* < 0.10,** *p* < 0.05,*** *p* < 0.01.

**Table 5:** Results of regression model (4)-(5)

	**(1)**	**(2)**	**(3)**	**(4)**	**(5)**	**(6)**
	**Standardization of physician’s prescription**			**Medication error recording and reporting**		
	**Group 1**	**Group 2**	**Group 3**	**Group 1**	**Group 2**	**Group 3**
Drug safety knowledge	0.192	0.0639	0.0465	0.218	0.217	0.128
	(1.37)	(0.42)	(0.40)	(0.82)	(0.93)	(0.71)
Drug safety attitude	0.111*	0.0701	0.0726	0.248*	0.107	0.0862
	(1.66)	(0.74)	(0.90)	(1.84)	(0.66)	(0.79)
Physical work environment	−0.0196	0.0677	−0.0200	−0.0412	−0.0714	0.00982
	(−0.56)	(1.21)	(−0.44)	(−0.72)	(−0.74)	(0.13)
Self-perceived pressure and fatigue	0.0821*	0.166***	0.344***	−0.0880	0.113	0.0572
	(1.71)	(3.15)	(4.59)	(−1.02)	(0.88)	(0.49)
Hospital management quality	−0.0475	−0.0170	−0.0255	0.0183	−0.117	0.0746
	(−1.21)	(−0.48)	(−0.57)	(0.29)	(−1.33)	(1.00)
Hospital regulation	0.116*	0.144*	0.171*	0.476***	0.538***	0.728***
	(1.82)	(1.87)	(1.90)	(4.61)	(4.18)	(4.69)
Hospital drug safety culture	0.371***	0.230**	−0.0000900	0.447**	0.259***	0.205
	(2.83)	(2.20)	(−0.00)	(2.47)	(2.66)	(1.17)
Supervisor’s work team management	0.0876	0.00741	0.0131	0.289**	0.157*	0.0154
	(1.51)	(0.10)	(0.19)	(2.18)	(1.66)	(0.13)
Cooperation atmosphere of work team	−0.0700	0.0642	0.0971	0.362***	0.257	−0.0461
	(−1.52)	(1.07)	(1.16)	(2.91)	(1.13)	(−0.32)
Drug safety culture of work team	−0.0225	0.119	0.0744	0.607***	0.490**	0.0645
	(−0.26)	(1.05)	(0.69)	(4.21)	(2.51)	(0.40)
Personal average working load dummy variables	Yes	Yes	Yes	Yes	Yes	Yes
Personal professional title dummy variables	Yes	Yes	Yes	Yes	Yes	Yes
Personal professional position dummy variables	Yes	Yes	Yes	Yes	Yes	Yes
Hospital level dummy variables	Yes	Yes	Yes	Yes	Yes	Yes
Length of hospital history dummy variables	Yes	Yes	Yes	Yes	Yes	Yes
Hospital type dummy variables	Yes	Yes	Yes	Yes	Yes	Yes
Constant	0.906	1.453*	0.589	−3.043**	−1.928	−1.289
	(1.12)	(1.94)	(0.90)	(−2.32)	(−1.49)	(−1.18)
N	777	511	671	777	511	671
R^2^	0.236	0.308	0.258	0.293	0.251	0.229

*t* statistics in parentheses, * *p* < 0.10,** *p* < 0.05,*** *p* < 0.01.

**Table 6:** Results of regression model (6)-(7)

	**(1)**	**(2)**	**(3)**	**(4)**	**(5)**	**(6)**
	**Medication error analysis, sharing and correction**			**Implementation of drug safety system and measure**		
	**Group 1**	**Group 2**	**Group 3**	**Group 1**	**Group 2**	**Group 3**
Drug safety knowledge	0.267*	0.183	0.0165	0.129*	0.0961	0.00376
	(1.66)	(1.32)	(0.09)	(1.87)	(1.12)	(0.04)
Drug safety attitude	0.358***	0.350***	0.368***	0.233***	0.167***	0.107**
	(4.44)	(3.19)	(3.03)	(5.84)	(3.14)	(2.16)
Physical work environment	0.102**	0.149**	0.159***	−0.0178	−0.0202	0.00407
	(2.42)	(2.31)	(3.02)	(−0.85)	(−0.64)	(0.15)
Self-perceived pressure and fatigue	−0.0410	−0.0548	0.0233	0.239***	0.242***	0.254***
	(−0.65)	(−0.63)	(0.29)	(7.64)	(5.71)	(5.93)
Hospital management quality	−0.0434	−0.0243	0.0110	0.0508*	0.0711***	0.0814***
	(−0.92)	(−0.41)	(0.42)	(1.76)	(2.97)	(3.04)
Hospital regulation	0.433***	0.455***	0.534***	0.102***	0.207***	0.280***
	(5.72)	(4.35)	(5.97)	(2.70)	(4.06)	(5.91)
Hospital drug safety culture	0.290**	0.0894	0.0371	0.254***	0.191***	−0.0423
	(2.21)	(1.25)	(0.54)	(5.85)	(4.46)	(−0.66)
Supervisor’s work team management	0.273**	−0.0281	−0.0919	0.174***	−0.0959	−0.0986
	(2.17)	(−0.31)	(−1.14)	(5.07)	(−1.63)	(−1.33)
Cooperation atmosphere of work team	0.395***	0.105	0.0979	0.387***	0.0618	0.0549
	(3.33)	(1.08)	(1.13)	(5.81)	(1.31)	(1.20)
Drug safety culture of work team	0.406***	0.122***	0.171	0.416***	0.376***	0.0593*
	(2.67)	(3.15)	(1.62)	(7.95)	(5.88)	(1.67)
Personal average working load dummy variables	Yes	Yes	Yes	Yes	Yes	Yes
Personal professional title dummy variables	Yes	Yes	Yes	Yes	Yes	Yes
Personal professional position dummy variables	Yes	Yes	Yes	Yes	Yes	Yes
Hospital level dummy variables	Yes	Yes	Yes	Yes	Yes	Yes
Length of hospital history dummy variables	Yes	Yes	Yes	Yes	Yes	Yes
Hospital type dummy variables	Yes	Yes	Yes	Yes	Yes	Yes
Constant	−1.928**	−0.971	−1.857**	0.101	−0.309	−0.987**
	(−2.00)	(−1.12)	(−2.45)	(0.21)	(−0.73)	(−2.46)
N	777	511	671	777	511	671
R^2^	0.390	0.433	0.396	0.596	0.751	0.638

*t* statistics in parentheses, * *p* < 0.10,** *p* < 0.05,*** *p* < 0.01.

For the aspect of drug management, hospital regulation and pharmacist’s drug safety knowledge were major influencing factors in all three groups, drug safety culture of work team was major influencing factor only in group 1, and hospital management quality had small positive influence on drug management in all three groups.

For the aspect of pharmacist’s prescription dispensing behavior, pharmacist’s drug safety knowledge, hospital regulation, and pharmacist’s drug safety attitude were major influencing factors in all three groups, self-perceived pressure and fatigue and hospital management quality also had significant positive influences on pharmacist’s prescription dispensing behavior in all three groups, while drug safety culture of work team, cooperation atmosphere of work team, hospital drug safety culture, and supervisor’s work team management were major influencing factors only in group 1.

For the aspect of standardization of physician’s prescription (special aspect since it needed the cooperation of doctor and pharmacist), hospital drug safety culture was major influencing factor in group 1, self-perceived pressure and fatigue was major influencing factor in group 3, and both of them were major influencing factors in group 2.

For the aspect of medication error recording and reporting, hospital regulation was major influencing factor in all three groups, drug safety culture of work team and hospital drug safety culture were major influencing factors only in group 1 and group 2, while cooperation atmosphere of work team and supervisor’s work team management were major influencing factors only in group 1.

For the aspect of medication error analysis, sharing and correction, hospital regulation, pharmacist’s drug safety attitude, and physical work environment were major influencing factors in all three groups, drug safety culture of work team was major influencing factor only in group 1 and group 2, while cooperation atmosphere of work team, hospital drug safety culture, and supervisor’s work team management were major influencing factors only in group 1.

For the aspect of implementation of drug safety system and measure, self-perceived pressure and fatigue, pharmacist’s drug safety attitude, and hospital regulation were major influencing factors in all three groups, and hospital management quality also had significant positive influence on implementation of drug safety system and measure in all three groups, drug safety culture of work team and hospital drug safety culture were major influencing factors only in group 1 and group 2, while cooperation atmosphere of work team and supervisor’s work team management were major influencing factors only in group 1.

## Discussion

### Main findings of this study

Regional inequity in pharmacist’s drug safety practice still existed in China, and pharmacist’s drug safety practice in regions with higher per capita GDP and more abundant medical resources was still better than that in regions with lower per capita GDP and less abundant medical resources. Generally speaking, major influencing factors for pharmacist’s drug safety practice could be divided into two categories: the first category consisted of pharmacist’s drug safety knowledge, drug safety attitude, self-perceived pressure and fatigue, hospital management quality, and hospital regulation, they were usually major influencing factors for pharmacist’s drug safety practice/detailed aspects of pharmacist’s drug safety practice in all districts of China; the second category consisted of hospital drug safety culture, supervisor’s work team management, cooperation atmosphere of work team, and drug safety culture of work team, they were usually major influencing factors for pharmacist’s drug safety practice/detailed aspects of pharmacist’s drug safety practice only in regions with higher per capita GDP and more abundant medical resources.

The main cause of the above findings was that major influencing factors for pharmacist’s drug safety practice in the first category were paid attention to all around China, which induced that the link between pharmacist’s drug safety practice and influencing factors in the first category had already been established in all regions of China; but influencing factors for pharmacist’s drug safety practice in the second category were paid special attention to only in regions with higher per capita GDP and more abundant medical resources, which induced that the link between pharmacist’s drug safety practice and influencing factors in the second category had been established only in regions with higher per capita GDP and more abundant medical resources, and such link didn’t exist in regions with lower per capita GDP and less abundant medical resources [9].

Among major influencing factors for pharmacist’s drug safety practice in the first category: hospital regulation, pharmacist’s drug safety knowledge, and pharmacist’s drug safety attitude were always the most important influencing factors for pharmacist’s drug safety practice in all regions of China; hospital regulation was major influencing factor for all detailed aspects of pharmacist’s drug safety practice all around China; and pharmacist’s drug safety knowledge was major influencing factor for both the aspect of drug management and the aspect of pharmacist’s prescription dispensing behavior in all regions of China; pharmacist’s drug safety attitude was major influencing factor for the aspect of pharmacist’s prescription dispensing behavior, the aspect of medication error analysis, sharing and correction, and the aspect of implementation of drug safety system and measure all around China; and self-perceived pressure and fatigue was major influencing factor for both the aspect of standardization of physician’s prescription and the aspect of implementation of drug safety system and measure in all regions of China; while hospital management quality had significant positive influence on the aspect of drug management, the aspect of pharmacist’s prescription dispensing behavior, and the aspect of implementation of drug safety system and measure all around China.

Among major influencing factors for pharmacist’s drug safety practice in the second category: only in regions with higher per capita GDP and more abundant medical resources, both drug safety culture of work team and cooperation atmosphere of work team were the most important influencing factors for pharmacist’s drug safety practice, and both hospital drug safety culture and supervisor’s work team management were the second most important influencing factors for pharmacist’s drug safety practice; hospital drug safety culture, supervisor’s work team management, cooperation atmosphere of work team, and drug safety culture of work team were major influencing factors for the aspect of pharmacist’s prescription dispensing behavior, the aspect of medication error recording and reporting, the aspect of medication error analysis, sharing and correction, and the aspect of implementation of drug safety system and measure only in regions with higher per capita GDP and more abundant medical resources; while only hospital drug safety culture was major influencing factor for the aspect of standardization of physician’s prescription in regions with higher per capita GDP and more abundant medical resources.

### What is already known on this topic

Previous studies separately showed that pharmacist’s drug safety knowledge, drug safety attitude, and some detailed aspects of drug safety environment (involving facility and equipment, regulation, personal pressure and fatigue, hospital management quality, hospital drug safety culture, and drug safety culture of work team) all had positive influences on pharmacist’s drug safety practice [23-39], but their influences on pharmacist’s drug safety practice haven’t been studied under the same framework, and then their relative importance in influencing pharmacist’s drug safety practice wasn’t clear, most importantly, regional differences of their relative importance in influencing pharmacist’s drug safety practice haven’t been studied before [9].

For detailed aspects of pharmacist’s drug safety practice, most previous studies only focused on pharmacist’s basic drug safety practice (involving drug management, pharmacist’s prescription dispensing behavior, and medication error recording and reporting) and its major influencing factors, while little study paid attention to pharmacist’s advanced drug safety practice (involving medication error analysis, sharing and correction, and implementation of drug safety system and measure) and its major influencing factors [9,23-27,29,30,32,33,35-39]. And there was no previous study which explored regional differences in major influencing factors for detailed aspects of pharmacist’s drug safety practice in China.

Most previous studies mentioned above obtained their conclusions only through qualitative analysis and authors’ experiences, while little study adopted strict quantitative analysis, and their conclusions were usually drawn on the basis of a small range of pharmacists, then the robustness and universality of their conclusions remained controversial [23,24,26-32,34-39], and their conclusions couldn’t be directly used for regional comparative study.

### What this study adds

The most important contribution of this study was that both China’s regional inequity in pharmacist’s drug safety practice and major influencing factors for pharmacist’s drug safety practice among different districts of China were studied under a systematic and comprehensive framework for the first time. The second most important contribution of this study was that in order to perform the strict quantitative analysis, this study carried out the large-scale national patient safety and medication error baseline survey for the first time in China. The third most important contribution was that major influencing factors for pharmacist’s drug safety practice/detailed aspects of pharmacist’s drug safety practice among different districts of China were found for the first time in China, and compared with previous studies, the robustness and universality of these findings were much better.

On the basis of major findings, in China’s future health delivery system reform, the following inspirations on regional differences in effective ways to promote patient safety and drug safety through promoting pharmacist’s drug safety practice were found: major influencing factors for pharmacist’s drug safety practice in the first category (involving pharmacist’s drug safety knowledge, drug safety attitude, self-perceived pressure and fatigue, hospital management quality, and hospital regulation) should be further promoted in order to promote pharmacist’s drug safety practice in all districts of China; major influencing factors for pharmacist’s drug safety practice in the second category (involving hospital drug safety culture, supervisor’s work team management, cooperation atmosphere of work team, and drug safety culture of work team) should be further promoted in order to promote pharmacist’s drug safety practice in regions with higher per capita GDP and more abundant medical resources; the link between pharmacist’s drug safety practice and major influencing factors in the second category should be gradually established in regions with lower per capita GDP and less abundant medical resources.

### Limitation of this study

Several limitations should be noted. First, the response rate of the national patient safety and medication error baseline survey was only 33.52%, and then the collected sample slightly deviated from the stratified sampling design, which may have slight influence on the above findings. In fact the deviation of the collected sample was that the percentage of pharmacists in level 3 hospitals was slightly higher than the percentage in stratified sampling design, while the percentage of pharmacists in level 2 hospitals was slightly lower than the percentage in stratified sampling design. Second, due to the fact that questionnaire for the survey was designed on the basis of questionnaire for hospital survey on patient safety culture, patient safety/drug safety situation in China, and pharmacy experts’ experiences, there may be other potential influencing factors for pharmacist’s drug safety practice which were not contained in this study. Third, pharmacist’s drug safety knowledge, drug safety attitude, and detailed aspects of drug safety environment interrelated with each other, which may affect their relative importance in influencing pharmacist’s drug safety practice/detailed aspects of pharmacist’s drug safety practice.

### Target audience

Findings in this study may prove useful for both pharmacists and regulators among different districts of China. Pharmacist in different district could find the corresponding effective way to promote her/his drug safety practice through promoting corresponding major influencing factors. Regulator in different district could implement the corresponding effective intervention for pharmacist’s drug safety practice through targeted laws, policies, regulations, and measures, for example, promoting hospital drug safety culture, supervisor’s work team management, cooperation atmosphere of work team, and drug safety culture of work team through targeted laws, policies, regulations, and measures could help promote pharmacist’s drug safety practice in regions with higher per capita GDP and more abundant medical resources, but they could only first help establish the link between pharmacist’s drug safety practice and hospital drug safety culture/supervisor’s work team management/cooperation atmosphere of work team/drug safety culture of work team in regions with lower per capita GDP and less abundant medical resources.

## Conclusion

This study found that pharmacist’s drug safety practice in regions with higher per capita GDP and more abundant medical resources was still better than that in regions with lower per capita GDP and less abundant medical resources, and then regional inequity in pharmacist’s drug safety practice still existed in China. Pharmacist’s drug safety knowledge, drug safety attitude, self-perceived pressure and fatigue, hospital management quality, and hospital regulation were major influencing factors for pharmacist’s drug safety practice in all districts of China, while only in regions with higher per capita GDP and more abundant medical resources, hospital drug safety culture, supervisor’s work team management, cooperation atmosphere of work team, and drug safety culture of work team were major influencing factors for pharmacist’s drug safety practice.

For China’s future health delivery system reform, inspirations on regional differences in effective ways to promote patient safety and drug safety through promoting pharmacist’s drug safety practice were found as follows: in all districts of China, promoting pharmacist’s drug safety knowledge, drug safety attitude, self-perceived pressure and fatigue, hospital management quality, and hospital regulation could help promote pharmacist’s drug safety practice, while only in regions with higher per capita GDP and more abundant medical resources, promoting hospital drug safety culture, supervisor’s work team management, cooperation atmosphere of work team, and drug safety culture of work team could help promote pharmacist’s drug safety practice. And in regions with lower per capita GDP and less abundant medical resources, the link between pharmacist’s drug safety practice and hospital drug safety culture/supervisor’s work team management/cooperation atmosphere of work team/drug safety culture of work team should also be gradually established.

## Competing interests

The author declares that he has no competing interests.

## Authors’ contributions

LT carried out the data collection, performed the statistical analysis, conceived and drafted the manuscript. LT also approved the final manuscript. All authors read and approved the final manuscript.
